# Prognostic value of post-percutaneous coronary intervention diastolic pressure ratio

**DOI:** 10.1007/s12471-022-01680-0

**Published:** 2022-04-07

**Authors:** K. Masdjedi, L. J. C. van Zandvoort, T. Neleman, I. Kardys, J. Ligthart, W. K. Den Dekker, R. Diletti, F. Zijlstra, N. M. Van Mieghem, J. Daemen

**Affiliations:** Department of Cardiology, Thoraxcenter, Erasmus Medical Centre, Rotterdam, The Netherlands

**Keywords:** Coronary physiology, Non-hyperaemic pressure ratio, Diastolic pressure ratio

## Abstract

**Aim:**

To evaluate the distribution of a generic diastolic pressure ratio (dPR) after angiographically successful percutaneous coronary intervention (PCI) and to assess its association with the 2‑year incidence of target vessel failure (TVF), defined as a composite of cardiac mortality, target vessel revascularisation, target vessel myocardial infarction and stent thrombosis.

**Methods:**

The dPR SEARCH study is a post hoc analysis of the prospective single-centre FFR-SEARCH registry, in which physiological assessment was performed after angiographically successful PCI in a total of 1000 patients, using a dedicated microcatheter. dPR was calculated offline with recently validated software in a subset of 735 patients.

**Results:**

Mean post-PCI dPR was 0.95 ± 0.06. Post-PCI dPR was ≤ 0.89 in 15.2% of the patients. The cumulative incidence of TVF at 2‑year follow-up was 9.4% in patients with a final post-PCI dPR ≤ 0.89 as compared to 6.1% in patients with a post-PCI dPR > 0.89 (adjusted hazard ratio [HR] for dPR ≤ 0.89: 1.53; 95% CI 0.74–3.13; *p* = 0.249). dPR ≤ 0.89 was associated with significantly higher cardiac mortality at 2 years; adjusted HR 2.40; 95% CI 1.01–5.68; *p* = 0.047.

**Conclusions:**

In a real-world setting, despite optimal angiographic PCI results, 15.2% of the patients had a final post-PCI dPR of ≤ 0.89, which was associated with a higher incidence of TVF and a significantly higher cardiac mortality rate.

**Supplementary Information:**

The online version of this article (10.1007/s12471-022-01680-0) contains supplementary material, which is available to authorized users.

## What’s new?

- The resting index diastolic pressure ratio (dPR) is an excellent alternative to the instantaneous wave-free ratio (iFR).
- Despite angiographically successful percutaneous coronary intervention (PCI), in a significant number of vessels the post-PCI dPR remains suboptimal (≤ 0.89).
- Post-PCI dPR is associated with clinical outcome at follow-up.

## Introduction

An increasing body of evidence supports the use of either fractional flow reserve (FFR) or the non-hyperaemic instantaneous wave-free ratio (iFR) for intracoronary physiological assessment of intermediate coronary artery lesions [1, 2]. Recently, a series of so-called non-hyperaemic pressure ratios (NHPRs) have been validated and proved to have a nearly perfect correlation to iFR, enhancing the adoption of general NHPRs in real-world clinical practice [3–5]. At the same time, the use of post-percutaneous coronary intervention (post-PCI) physiological assessment is gaining attention. A strong and linear association has been demonstrated between post-PCI FFR and the risk for both future repeat revascularisation as well as hard clinical endpoints such as death and myocardial infarction [6–8]. The relevance of the latter was strengthened by recent work by our group demonstrating that post-PCI FFR values were < 0.90 in up to 37.8% of stented vessels despite optimal angiographic results [9]. With respect to post-PCI NHPRs, the recently published DEFINE PCI study showed that 22.6% of treated vessels had a final post-PCI iFR ≤ 0.89 [10].

To date, limited data are available on the distribution of post-PCI NHPRs and their prognostic value. The aim of the present study was to evaluate the distribution of a recently validated generic diastolic pressure ratio (dPR) after angiographically successful PCI in an all-comers study population and to study its association with 2‑year clinical outcome.

## Methods

### Study design and patient population

**Fig. 1:**
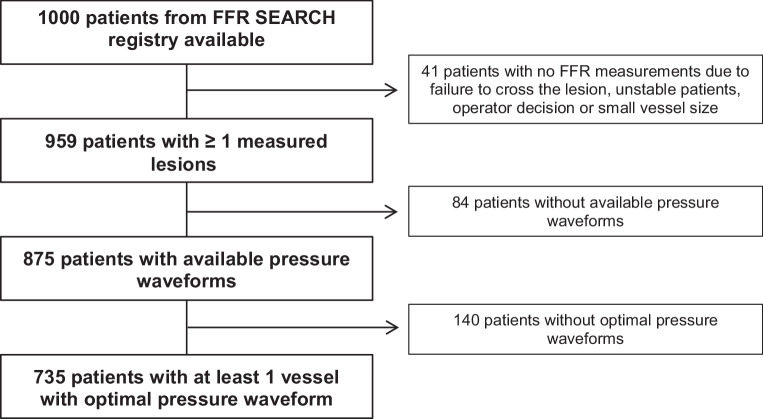
Flowchart showing all included and excluded patients

The dPR SEARCH study was a post hoc analysis of the FFR-SEARCH registry (Stent Evaluated at Rotterdam Cardiology Hospital), a prospective single-centre registry in which routine FFR measurements were performed after angiographically successful PCI in a total of 1000 patients between March 2016 and May 2017 [9]. Exclusion criteria were: (1) patients presenting with cardiogenic shock, (2) ‘high-risk’ procedures defined as use of mechanical circulatory support, (3) age < 18 years and (4) an estimated vessel size < 2.25 mm. A total of 735 patients (735 vessels) with available undamped pressure waveform data were selected for the present study (Fig. 1).

The study was performed in accordance with the Declaration of Helsinki. The study protocol was approved by the local ethics committee of the Erasmus Medical Centre. Participants were informed about the study by the physician responsible for the procedure and provided informed consent for the procedure and the use of anonymous datasets for research purposes in alignment with the Dutch Medical Research Act.

### Coronary angiography and calculation of FFR

All procedures were performed according to standard local clinical practice with the use of intracoronary imaging and physiology at the operator’s discretion. All vessels, including in-stent restenosis cases, were treated with a stent. Comprehensive quantitative coronary angiography analyses were performed pre- and post-stent implantation in all treated lesions. An angiographic view with minimal foreshortening of the lesion and minimal overlap with other vessels was selected. Similar angiographic views were used pre- and post-stent implantation. Measurements included: pre- and post-procedural percentage diameter stenosis; reference vessel diameter; lesion length and minimal luminal diameter (MLD). In patients with a total occlusion (those presenting with ST-elevation myocardial infarction [STEMI] or a chronic total occlusion [CTO]), the MLD was considered zero and percentage diameter stenosis 100%. Reference vessel diameter and lesion length were calculated from the first angiographic view with restored flow. All angiographic measurements were performed using CAAS for Windows, version 2.11.2 (Pie Medical Imaging, Maastricht, The Netherlands).

Pressure measurements were performed after an intracoronary bolus of nitrates (100–200 µg) using a dedicated rapid exchange monorail microcatheter (Navvus RXi system; ACIST Medical Systems Inc., Eden Prairie, MN, USA), with a fibre-optic-based sensor technology compatible with standard 0.014-inch guidewires [11, 12]. After equalisation of the system based on undampened pressure waveforms, the device was inserted over the previously used coronary guidewire approximately 20 mm distal to the most distal stent edge, at which point *P*_d_/*P*_a_ was measured. FFR values were subsequently recorded at four different positions in the coronary artery: (1) 20 mm distal to the distal stent edge, (2) at the distal stent edge, (3) at the proximal stent edge and (4) at the coronary ostium to verify the occurrence of drift. In cases of significant drift (≥ 3 units) measurements were repeated. All analyses performed in the present study were based on values measured 20 mm distal to the most distal stent edge. In patients in which dPR was assessed in multiple vessels, only the vessel with the lowest dPR was included.

### Definition and calculation of dPR

*P*_d_/*P*_a_ was defined as the ratio of mean distal coronary artery pressure to mean aortic pressure in the resting state during the whole cardiac cycle. FFR was defined as the lowest ratio of mean distal coronary artery pressure divided by mean aortic pressure during maximal hyperaemia. dPR was defined as the ratio between the mean diastolic pressure distal to the stenosis and the mean diastolic aortic pressure in resting conditions, taken over an average of 5 consecutive heartbeats, based on the initial distal resting *P*_d_/*P*_a_. The dPR was calculated retrospectively using recently validated dedicated software developed at the Erasmus Medical Centre [3]. Briefly, the diastolic period used to calculate the dPR was automatically delineated based on the d*P*/d*t* curve of the aortic pressure at the point at which the resistance was low, constant and stable. The d*P*/d*t* curve represents the increase and decrease of the pressure over time during the heart cycle. d*P* is the pressure difference between sample points and d*t* is the time difference between the same sample points.

### Endpoint definitions and clinical follow up

The primary endpoint consisted of target vessel failure (TVF), defined as a composite of cardiac mortality, target vessel revascularisation (TVR), target vessel myocardial infarction (TVMI) and stent thrombosis (ST) at 2 years. Secondary endpoints included the individual components of the primary endpoint and all-cause mortality. Clinical follow-up data were obtained from electronic medical records of the hospital and general practitioner. Survival data were obtained from the municipal civil registry. In addition, all surviving patients were contacted in person or by telephone with specific queries on clinical outcome. Cardiac mortality was defined as any death due to a proximate cardiac cause, unwitnessed death or death of unknown cause [13]. Myocardial infarction was diagnosed in accordance with the expert consensus document, defined as a rise and/or fall of troponin with at least one value above the 99th percentile of the upper reference limit together with evidence of myocardial ischaemia with at least one of the following: (1) symptoms of ischaemia, (2) electrocardiographic changes indicative of new ischaemia (new ST-segment and T‑wave changes or new left bundle branch block), (3) development of pathological waves in the electrocardiogram and (4) imaging evidence of new loss of viable myocardium or new regional wall motion abnormality [14, 15]. ST was defined as angiographically defined thrombosis within the stent or 5 mm proximal or distal to the stent with the presence of a flow-limiting thrombus, accompanied by acute symptoms. Event adjudication was performed by trained study personnel unaware of the final physiological assessment.

### Statistical analysis

Baseline, categorical variables are reported as either counts or percentages and reported as mean ± standard deviation. The association between dPR and clinical endpoints was analysed by Cox proportional hazard regression analysis. Univariate predictors of outcomes were identified using the Cox proportional-hazards model. Clinically relevant variables (age, male gender, diabetes mellitus and STEMI at presentation) were introduced into the multivariate Cox proportional-hazards model using the ‘enter’ method. Data are presented as hazard ratio (HR) with a 95% confidence interval (95% CI). All tests were two-tailed and a *p*-value < 0.05 was considered statistically significant. The Kaplan-Meier method was applied to show the cumulative incidence of the primary and secondary endpoints, whereas log-rank tests were used to evaluate differences between the groups. Patients that were lost to follow-up were censored at the date of the last contact. Receiver-operating characteristic (ROC) curve analysis was performed to assess the optimal cut-off value of post-PCI dPR for predicting clinical outcome. However, due to the limited number of events, the ROC curve was not able to identify a final post-PCI dPR value to predict TVF (Electronic Supplementary Material, Fig. S1). Given the exploratory nature of the present study, we deliberately took the accepted ischaemic dPR threshold of 0.89 as a cut-off value to predict clinical outcome. A predefined subgroup analysis was performed in patients presenting with stable- or unstable angina or non-STEMI (NSTEMI). Statistical analyses were performed using SPSS statistics for Windows, version 24.0 (SPSS, Chicago, IL, USA).

## Results

### Patient demographics and procedural data

A total of 735 patients (735 vessels) were included. Patients’ baseline characteristics are depicted in Tab. 1. In brief, 75% of the patients were male and average age was 64 ± 12 years. Hypertension was present in 51% of the cases and 19% were diabetic. Clinical presentation was stable angina in 31% of the cases, whereas 36% and 33% of the patients presented with NSTEMI and STEMI, respectively. Vessel and lesion characteristics are presented in Tab. 2.

**Table 1:** Patients’ baseline characteristics (*n* = 735)

	Total (*n* = 735)	dPR ≤ 0.89 (*n* = 112)	dPR > 0.89 (*n* = 623)	*p*-value
Age (years), mean ± SD	64 ± 12	65 ± 11	64 ± 12	0.381
Male gender, *n* (%)	552 (75)	90 (80)	462 (74)	0.162
*Cardiovascular risk factors, n (%)*				
Hypertension	373 (51)	70 (63)	303 (49)	0.006
Hypercholesterolaemia	329 (45)	58 (52)	271 (44)	0.114
Diabetes mellitus	140 (19)	37 (33)	103 (17)	< 0.001
Smoking history	368 (50)	43 (38)	325 (52)	0.007
Peripheral artery disease	52 (7)	9 (8)	43 (7)	0.667
*Cardiovascular comorbidity, n (%)*				
Prior stroke	51 (7)	7 (6)	44 (7)	0.755
Prior myocardial infarction	144 (20)	24 (21)	120 (19)	0.595
Prior PCI	192 (26)	30 (27)	162 (26)	0.862
Prior CABG	42 (6)	5 (5)	37 (6)	0.536
Haemoglobin (mmol/l), mean ± SD	8.7 ± 1.0	8.5 ± 1.1	8.70 ± 1.0	0.095
Creatinine (µmol/l), mean ± SD	93 ± 53	107 ± 99	90 ± 38	0.001
*Indication, n (%)*				
Stable angina	231 (31)	41 (37)	190 (31)	0.200
NSTEMI	263 (36)	46 (41)	217 (35)	0.205
STEMI	241 (33)	25 (22)	216 (35)	0.010

*dPR* diastolic pressure ratio, *PCI* percutaneous coronary intervention, *CABG* coronary artery bypass grafting, *STEMI* ST-segment elevation myocardial infarction, *NSTEMI* non-STEMI

**Table 2:** Vessel and lesion characteristics (*n* = 735)

	Total (*n* = 735)	dPR ≤ 0.89 (*n* = 112)	dPR > 0.89 (*n* = 623)	*p*-value
dPR (mean ± SD)	0.95 ± 0.06	0.86 ± 0.04	0.97 ± 0.04	< 0.001
*Lesion type, n (%)*				
A	70 (10)	5 (5)	65 (10)	0.048
B1	156 (21)	19 (17)	137 (22)	0.231
B2	232 (31)	46 (41)	186 (30)	0.019
C	277 (38)	42 (38)	235 (38)	0.965
*Measured vessels, n (%)*				
Left main	17 (2)	4 (4)	13 (2)	0.336
Left anterior descending artery	383 (52)	98 (88)	285 (46)	< 0.001
Left circumflex artery	125 (17)	5 (5)	120 (19)	< 0.001
Right coronary artery	204 (28)	5 (5)	199 (32)	< 0.001
Vein graft	6 (1)	0 (0)	6 (1)	0.297
*Lesion characteristics, n (%)*				
Bifurcation	85 (12)	20 (18)	65 (11)	0.024
Moderate to severe calcification	268 (37)	55 (49)	213 (34)	0.003
In-stent restenosis	22 (3)	4 (4)	18 (3)	0.696
Thrombus	142 (19)	13 (12)	129 (21)	0.025
Stent thrombosis	9 (2)	1 (1)	8 (1)	0.729
Ostial	73 (10)	10 (9)	63 (10)	0.700
CTO	30 (4)	10 (9)	20 (3)	0.005
Pre-dilatation	501 (68)	88 (79)	413 (66)	0.010
Post-dilatation	455 (62)	77 (69)	378 (61)	0.109
* 2D-QCA measurements (mean* *±* *SD)*				
Stenosis pre, %	65 ± 22	61 ± 22	65 ± 22	0.052
Stenosis post, %	4 ± 13	3 ± 15	4 ± 13	0.585
Ref diameter pre, mm	2.7 ± 0.6	2.5 ± 0.6	2.7 ± 0.6	< 0.001
Ref diameter post, mm	2.8 ± 0.5	2.5 ± 0.5	2.8 ± 0.5	< 0.001
Length pre, mm	21 ± 12	21 ± 11	21 ± 12	0.983
Length post, mm	24 ± 14	25 ± 12	24 ± 14	0.809
MLD pre, mm	0.94 ± 0.6	0.95 ± 0.6	0.94 ± 0.6	0.928
MLD post, mm	2.6 ± 0.5	2.4 ± 0.4	2.7 ± 0.5	< 0.001

*dPR* diastolic pressure ratio, *CTO* chronic total occlusion, *QCA* quantitative coronary angiography, *MLD* minimum lumen diameter

### Distribution of dPR and clinical outcome at 2 year follow up

**Fig. 2:**
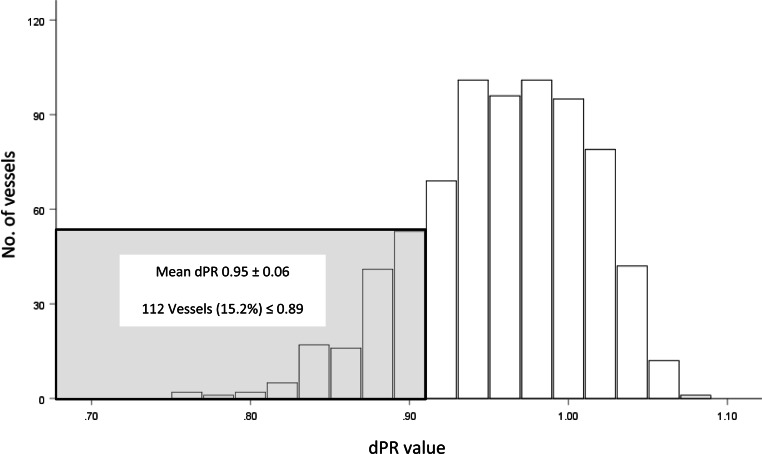
Distribution of post-percutaneous coronary intervention diastolic pressure ratio (*dPR*)

**Fig. 3:**
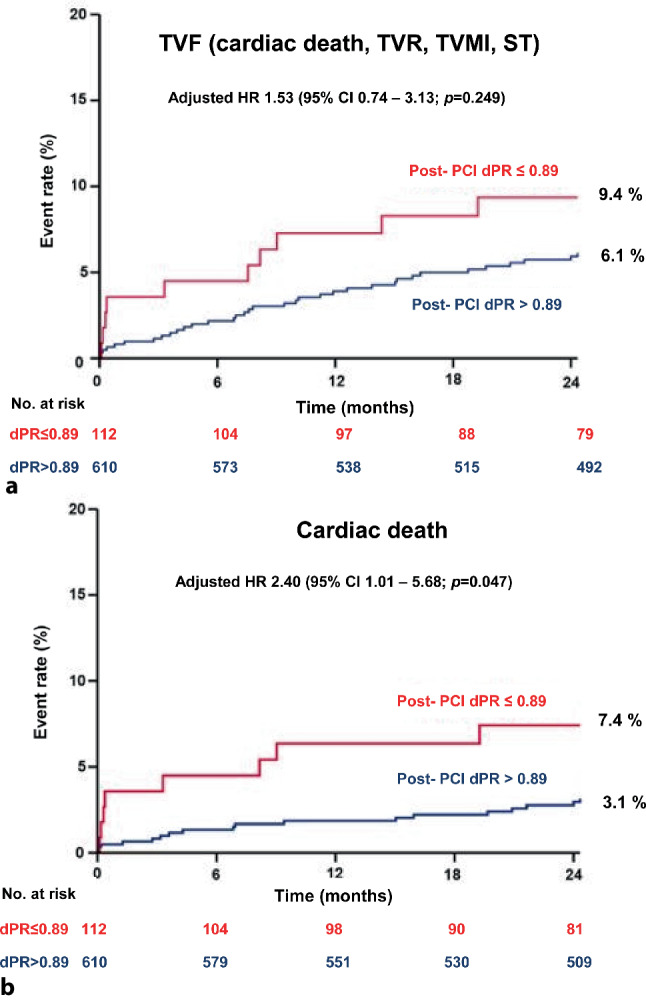
Cumulative incidence of target vessel failure (*TVF*) and cardiac death at 2‑year follow-up. (*TVR* target vessel revascularisation, *TVMI* target vessel myocardial infarction, *ST* stent thrombosis, *HR* hazard ratio, *CI* confidence interval, *PCI* percutaneous coronary intervention, *dPR* diastolic pressure ratio)

Mean post-PCI dPR was 0.95 ± 0.06. Mean drift was 0.01 ± 0.01. Post-PCI dPR was ≤ 0.89 in 15.2% of the cases (Fig. 2). The cumulative incidence of TVF was 6.1% in patients with a final post-PCI dPR ≤ 0.89 as compared to 9.4% in patients with a post-PCI dPR > 0.89 [adjusted HR for dPR ≤ 0.89: 1.53; 95% CI 0.74–3.13; *p* = 0.249]. Cardiac mortality rates were significantly higher in patients with a final post-PCI dPR ≤ 0.89 as compared to those with a dPR > 0.89 [7.4% vs 3.1%, adjusted HR 2.40, 95% CI 1.01–5.68; *p* = 0.047] (Fig. 3; Tab. 3; and Electronic Supplementary Material, Tab. S1).

**Table 3:** Cumulative incidence of clinical outcome at 2‑year follow-up

	Total (*n* = 735) *n* (%)	dPR ≤ 0.89 (*n* = 112) %	dPR > 0.89 (*n* = 623) %	Log-rank *p*-value
All-cause mortality	43 (5.9)	10.3	5.2	0.033
Cardiac mortality^a^	26 (3.5)	7.4	3.1	0.023
TVR	19 (2.6)	2.0	3.0	0.566
TVMI	16 (2.2)	4.0	2.1	0.267
ST	10 (1.4)	0.9	1.6	0.644
TVF^a^	45 (6.1)	9.4	6.1	0.176

*dPR* diastolic pressure ratio, *TVR* target vessel revascularisation, *TVMI* target vessel myocardial infarction, *ST* stent thrombosis, *TVF* target vessel failure

^a^TVR, TVMI and ST

### Stratified analysis in patients presenting with stable or unstable angina or NSTEMI

A total of 494 patients (67.2%) presented with stable- or unstable angina or NSTEMI. The cumulative incidence of TVF was 11.8% in patients with a final post-PCI dPR ≤ 0.89 as compared to 6.5% in patients with a post-PCI dPR > 0.89 [adjusted HR for dPR ≤ 0.89: 1.92; 95% CI 0.91–4.01; *p* = 0.070] (Electronic Supplementary Material, Fig. S2).

## Discussion

In the present study focusing on the real world impact of post-PCI dPR, we demonstrated that: (1) despite optimal angiographic results 15.2% of the vessels have a final post-PCI dPR of ≤ 0.89; (2) the incidence of TVF was higher in patients with post-PCI dPR ≤ 0.89; however, (3) a post-PCI dPR ≤ 0.89 was associated with a higher cardiac mortality rate.

Despite the unequivocal evidence supporting the use of pre-PCI physiological lesion assessment, the use of the technology in a post-PCI setting is still rare. Instead, post-PCI results are routinely assessed by visual angiographic assessment, a technique that has repeatedly been shown to correlate poorly with invasive functional assessment [16–18]. The importance of the latter is further illustrated by a growing body of evidence showing the strong predictive value of post-PCI FFR for future adverse events [19–22]. However, little is known about the use of post-PCI dPR and its predictive value. To the best of our knowledge, the present study is the largest on the distribution and predictive value of post-PCI dPR to date and the first to assess the correlation between post-PCI dPR and 2‑year clinical outcome.

We were able to demonstrate that in an all-comers study population, despite satisfactory angiographic results, 15.2% of the patients had a post-PCI dPR ≤ 0.89. Our work thereby complements the findings of the DEFINE PCI study, in which 22.6% of the treated vessels had a final post-PCI iFR ≤ 0.89 [10]. Our work, however, differed from the DEFINE PCI study by enrolling a larger and more real-world patient population, in which patients with prior coronary artery bypass graft, CTO treatment, STEMI and TIMI flow < 3 were not excluded. Especially the inclusion of patients presenting with STEMI and the lower number of patients with diabetes (19% vs 34%, respectively) might explain the lower number of patients with a post-PCI dPR ≤ 0.89 as compared to those in the DEFINE PCI study [23]. Despite the restoration of epicardial flow through PCI, patients with STEMI have abnormal myocardial perfusion at the end of the procedure [24]. This phenomenon is thought to be related to microvascular obstruction due to distal embolisation, reperfusion injury and tissue inflammation due to myocyte necrosis [25]. In addition, physiological assessment in patients with diabetes mellitus underestimates the disease severity because of diffuse coronary atherosclerosis, microvascular disease and a tendency for negative remodelling [26]. The last-mentioned resulted in the pre-defined subanalysis in patients presenting with stable or unstable angina or NSTEMI, in which a more pronounced effect of post-PCI dPR ≤ 0.89 was seen to predict 2‑year TVF rates.

In the present study pressure measurements were performed approximately 20 mm distal to the distal stent edge, while in the DEFINE PCI study the investigators reported consistently placing their pressure sensors in the distal third of the study vessel, which is another potential explanation for the lower proportion of patients with dPR ≤ 0.89 in the present study.

Despite a growing body of evidence on the strong correlation between post-PCI FFR and the risk for future adverse cardiovascular events, the present study is the first to assess the correlation between post-PCI dPR and clinical outcome at 2 years [21, 22]. We found a higher incidence of TVF in patients with post-PCI dPR ≤ 0.89 as compared to those with a dPR > 0.89. More specifically, a post-PCI dPR ≤ 0.89 proved to be associated with a 2.4-fold increased risk for cardiac mortality at 2 years when corrected for clinically relevant variables such as age, gender, diabetes mellitus and STEMI at presentation (*p* = 0.047). Association of low post-PCI dPR and increased risk for cardiac mortality is in line with the results of the recently presented 1‑year follow-up of the DEFINE PCI study, which showed that post-PCI iFR < 0.95 was associated with lower event rates [27].

The microcatheter-based FFR system has a small lumen profile (maximum diameter 0.036 inch at sensor level and 0.025 inch at optical-fibre level) offering some potential advantages as compared to the conventional pressure-wire-based systems, including easy delivery over routine coronary guidewires and the use of a fibre-optic sensor that proved to significantly reduce drift as compared to conventional pressure wires with piezoelectric sensors [28]. Conversely, previous studies demonstrated that microcatheter-based systems may slightly overestimate stenosis severity as compared to the conventional wire-based FFR systems. The mean overall bias between microcatheter FFR and conventional wire-based FFR was −0.029 (microcatheter FFR was lower), a bias that proved mostly relevant in the case of lower FFR values or small-calibre vessels [29, 30]. In the present study the measurements were performed after angiographically successful stenting. Subsequent mean FFR values were therefore relatively high (mean 0.90) and significantly higher as compared to, for instance, the mean FFR values in the ACIST-FFR study (FFR 0.81) [28]. Therefore, the authors believe that in the present study the impact of smaller luminal diameters on the bias between microcatheter- and pressure-wire-based values was limited.

The present study demonstrates the feasibility of post-PCI physiological assessment using a dedicated monorail microcatheter without the need for hyperaemic agents associated with increased time, costs and side-effects. Routine physiological post-PCI dPR assessment identifies a significant number of patients with suboptimal post-PCI results that are at increased risk for future adverse cardiac events. The ongoing randomised FFR-REACT trial will assess whether invasive imaging and PCI optimisation (using additional stents and post-dilation) will improve outcomes in patients with suboptimal post-PCI physiological measurements [31].

### Limitations

Several limitations deserve to be mentioned. First of all, post-physiological assessment was performed using the Navvus microcatheter, which is an over-the-wire microcatheter with a profile of 0.022 inch that resulted in a slightly but significantly lower FFR (by 1–3%) as compared to the conventional 0.014-inch pressure wires [32]. In addition, the results are based on a single-centre experience in which we restricted our analyses to recordings with adequate pressure waveforms. The latter could have artificially influenced our results, since previous work, assessing the prevalence of erroneous or suboptimal FFR measurements in clinical practice, demonstrated that in up to 30% of the recordings, pressure signals were inadequate [33]. Furthermore, due to the nature of our real-world registry, in which most patients were referred for PCI by satellite hospitals, left ventricular (LV) function was missing in a considerable number of cases. Given the high number of missing values we decided to refrain from adjusting our findings for LV function. Finally, the data acquisition protocol of the FFR-SEARCH registry included only a pullback during maximum hyperaemia, precluding us from analysing detailed post-procedural dPR gradients within the treated vessel.

## Conclusion

Despite optimal angiographic PCI results, 15.2% of the patients had a final post-PCI dPR of ≤ 0.89, which was associated with a significantly higher cardiac mortality rate. The incidence of TVF was higher in patients with post-PCI dPR ≤ 0.89.

## Supplementary Information

Fig. S1 Poor ability of ROC curve to identify a final post-PCI dPR value to predict TVF

Table S1 Association of post PCI dPR and risk of clinical events at 2 year follow up

Fig. S2 Cumulative incidence of target vessel failure (Cardiac death, TVR, TVMI, ST) in patients without SETMI. *HR* hazard ratio, *CI* confidence interval, *PCI* percutaneous coronary intervention, *dPR* diastolic pressure ratio.
