# Supplementary figures and images for: Prognostic value of post-percutaneous coronary intervention diastolic pressure ratio
Source: Neth Heart J. 2022 Apr 7;30(7-8):352–9. doi: 10.1007/s12471-022-01680-0 (PMC9270544; doi:10.1007/s12471-022-01680-0)

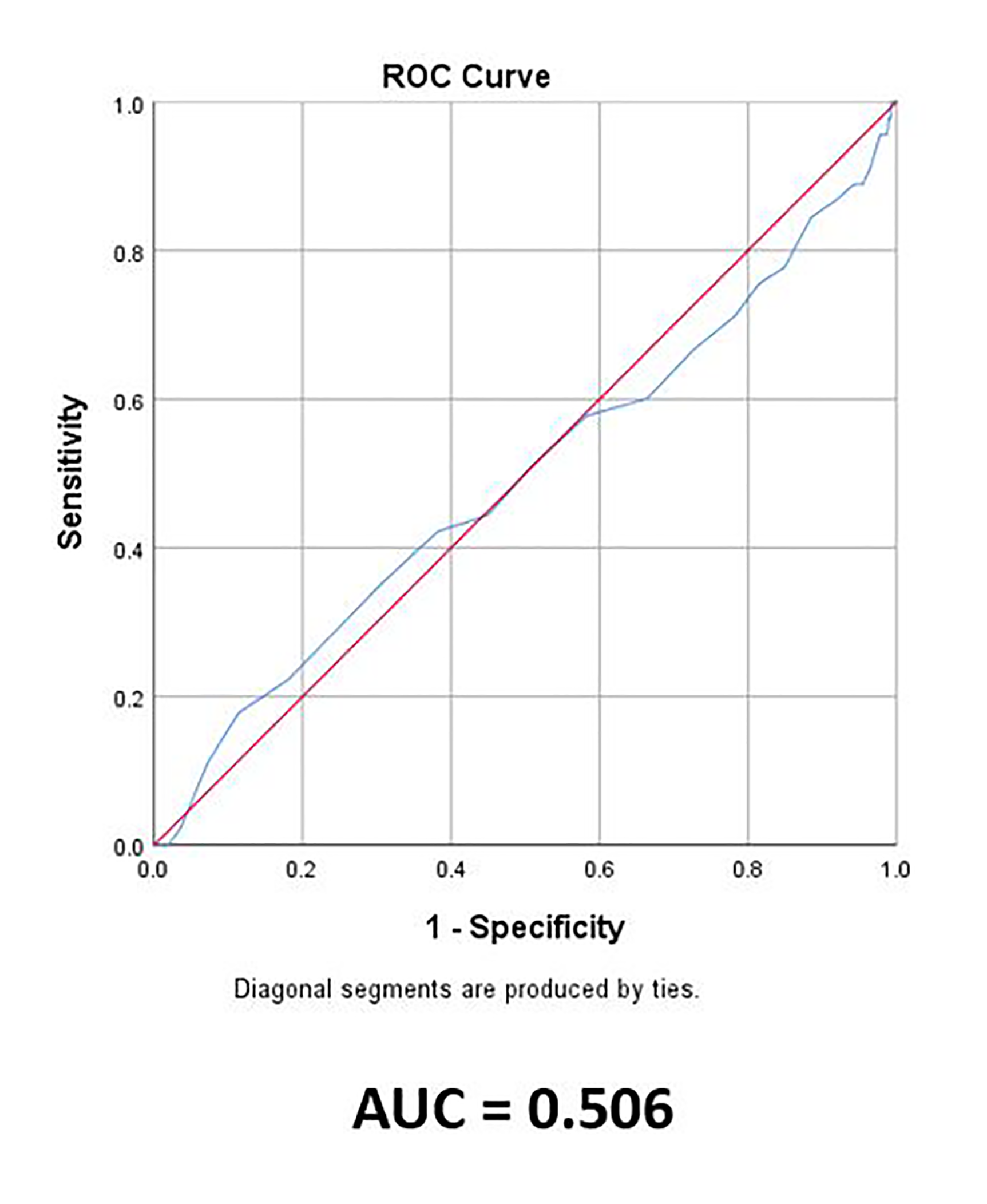

Supplement: Supplementary file 1 — Fig. S1 Poor ability of ROC curve to identify a final post-PCI dPR value to predict TVF [file 12471_2022_1680_MOESM1_ESM.tif]

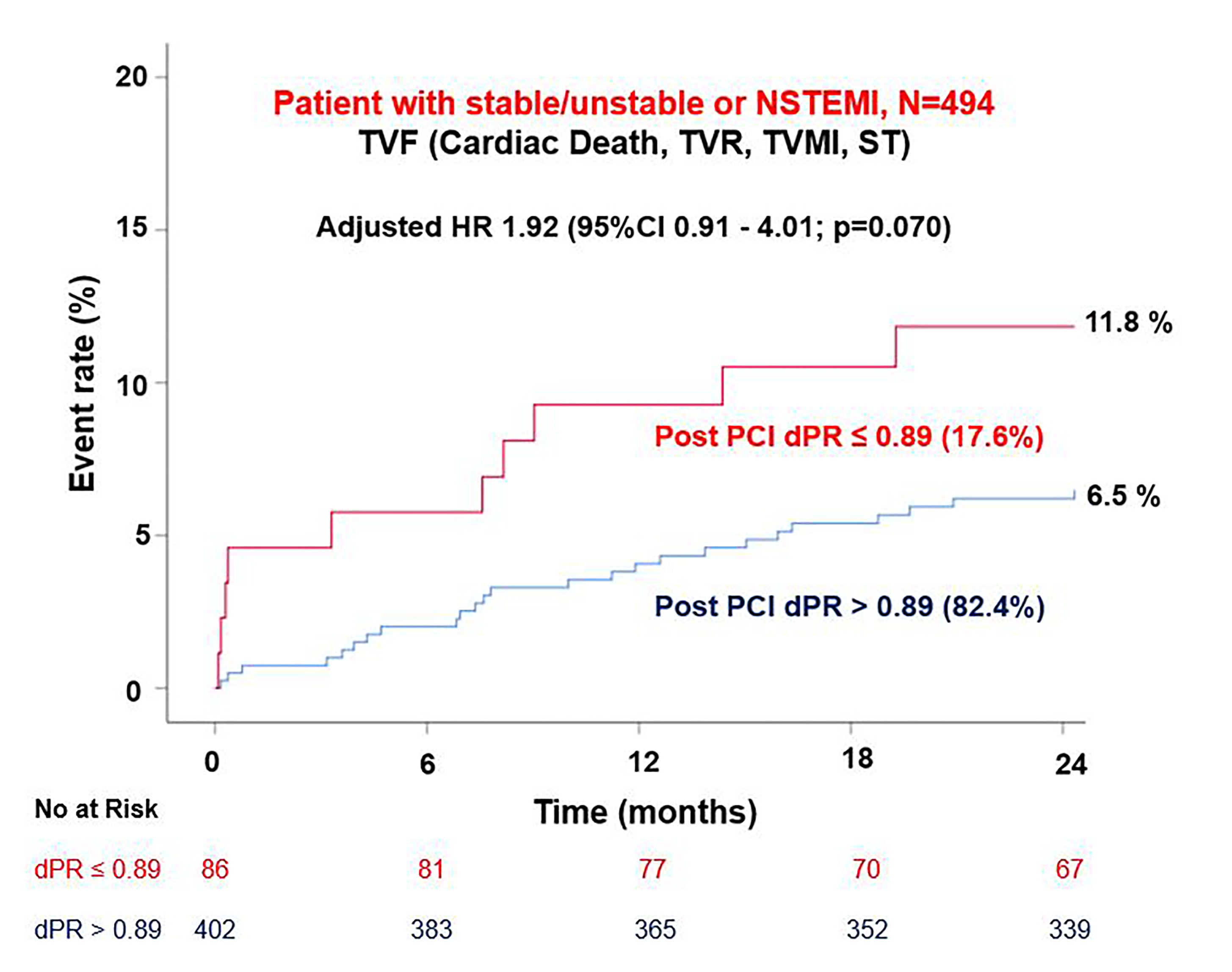

Supplement: Supplementary file 3 — Fig. S2 Cumulative incidence of target vessel failure (Cardiac death, TVR, TVMI, ST) in patients without SETMI. HR hazard ratio, CI confidence interval, PCI percutaneous coronary intervention, dPR diastolic pressure ratio. [file 12471_2022_1680_MOESM3_ESM.tif]
