# Supplementary material for: Prognostic value of post-percutaneous coronary intervention diastolic pressure ratio
Source: Neth Heart J. 2022 Apr 7;30(7-8):352–9. doi: 10.1007/s12471-022-01680-0 (PMC9270544; doi:10.1007/s12471-022-01680-0)
Supplement: Supplementary file 2 — Table S1 Association of post PCI dPR and risk of clinical events at 2 year follow up [file 12471_2022_1680_MOESM2_ESM.docx]

**Table S1.** Association of post PCI dPR and risk of clinical events at 2 year follow up

|  | **Univariable**  **HR (95% CI)** | **p- value** | **Multivariable**  **HR (95% CI)** | **p- value** |
| --- | --- | --- | --- | --- |
| **Target Vessel Failure (TVF)** | | | | |
| Post PCI dPR≤0.89 | 1.62 (0.80-3.26) | 0.181 | 1.53 (0.74-3.13) | 0.249 |
| Age | 1.02 (0.99-1.05) | 0.128 | 1.02 (0.99-1.05) | 0.199 |
| Male gender | 0.82 (0.43-1.56) | 0.542 | 0.87 (0.45-1.69) | 0.684 |
| Diabetes Mellitus | 1.23 (0.61-2.48) | 0.570 | 1.09 (0.53-2.24) | 0.809 |
| STEMI | 0.636 (0.32-1.29) | 0.207 | 0.70 (0.34-1.42) | 0.320 |
| **Cardiac Mortality** | | | | |
| Post PCI dPR≤0.89 | 2.53 (1.10-5.83) | 0.029 | 2.40 (1.01-5.68) | 0.047 |
| Age | 1.05 (1.01-1.08) | 0.012 | 1.05 (1.01-1.09) | 0.015 |
| Male gender | 0.90 (0.38-2.15) | 0.816 | 1.04 (0.43-2.53) | 0.936 |
| Diabetes Mellitus | 1.30 (0.52-3.23) | 0.579 | 1.14 (0.45-2.91) | 0.783 |
| STEMI | 0.826 (0.35-1.96) | 0.665 | 1.00 (0.41-2.43) | 0.996 |
| **Target Vessel Revascularization (TVR)** | | | | |
| Post PCI dPR≤0.89 | 0.65 (0.15-2.83) | 0.569 | 0.64 (0.15-2.83) | 0.560 |
| Age | 0.99 (0.96-1.03) | 0.748 | 0.99 (0.95-1.03) | 0.534 |
| Male gender | 0.72 (0.27-1.89) | 0.505 | 0.72 (0.27-1.93) | 0.516 |
| Diabetes | 0.80 (0.23-2.73) | 0.718 | 0.73 (0.21-2.53) | 0.619 |
| STEMI | 0.26 (0.06-1.14) | 0.073 | 0.24 (0.06-1.06) | 0.059 |
| **Target Vessel Myocardial Infarction (TVMI)** | | | | |
| Post PCI dPR≤0.89 | 1.88 (0.61-5.82) | 0.275 | 1.92 (0.61-6.10) | 0.268 |
| Age | 0.99 (0.95-1.03) | 0.696 | 0.98 (0.94-1.03) | 0.414 |
| Male gender | 0.56 (0.20-1.53) | 0.257 | 0.52 (0.19-1.44) | 0.206 |
| Diabetes | 0.99 (0.28-3.46) | 0.983 | 0.79 (0.22-2.83) | 0.713 |
| STEMI | 0.32 (0.07-1.39) | 0.128 | 0.31 (0.07-1.40) | 0.129 |
